# Leber’s Hereditary Optic Neuropathy – Case Discussion

**Published:** 2019

**Authors:** Cristina Culea, Bogdana Tăbăcaru, Simona Stanca, Horia Tudor Stanca

**Affiliations:** * “Prof. Dr. Agrippa Ionescu” Clinical Emergency Hospital, Bucharest, Romania; ** “Carol Davila” University of Medicine and Pharmacy Bucharest, Romania

**Keywords:** absence of relative afferent pupillary defect, idebenone, maternal inheritance, pattern of nerve fiber damage, selective vulnerability of the optic nerve and the retinal ganglion cells

## Abstract

**Purpose.** To report a case of a young patient with a clinical condition suggestive of Leber’s hereditary optic neuropathy (LHON) confirmed by genetic testing.

**Material and methods.** We present a case of a 21-year-old Caucasian male with bilateral visual loss. The patient complained of visual loss, initially in the right eye and two weeks thereafter in the left eye. Ophthalmological examination revealed visual acuity of 20/ 400 in both eyes, anterior segment of normal appearance, normal direct and consensual pupillary light reflexes, and absence of a relative afferent pupillary defect. Fundus examination demonstrated bilateral protruding, hyperemic, with blurred margins in the nasal quadrant papilla and reduced excavation, tortuous vessels, peripapillary telangiectasias. The optical coherence tomography (OCT) revealed bilateral increase of the retinal nerve fiber layer (RNFL) thickness and ganglion cell layer – inner plexiform layer complex (GCL-IPL complex) severely thinned.

**Results.** The clinical suspicion of Leber’s hereditary optic neuropathy was confirmed by the 3460 mutation, which was identified on blood mitochondrial analysis. Meantime, the visual acuity decreased to CF in both eyes. We initiated treatment with idebenone (300 mg T.I.D.). After three months of follow-up, visual acuity was CF in both eyes, bilateral pupillary light reflexes within normal limits and optic disc pallor was noticed in both eyes.

**Conclusion.** No visual recovery was noticed after one year. We recommended that the idebenone treatment was continued and the patient was followed-up further.

## Introduction

LHON is caused by a variety of maternal transmission mutations with variable penetration and it is characterized by bilateral, painless, acute visual failure in one eye that develops during young adult life. Males are four to five times more likely than females to be affected, but neither gender nor mutational status significantly influences the timing and severity of the initial visual loss. Similar symptoms appear in the other eye on an average of two to three months later. Fundus examination features disk hyperemia, edema of the peripapillary retinal nerve fiber layer, retinal telangiectasia, and increased vascular tortuosity. The most important characteristic feature is an enlarging central or centrocecal scotoma and as the field defect increases in size and density, visual acuity deteriorates to the level of counting fingers or worse. After the acute phase, the optic discs become atrophic [**1**]. Regarding risk factors, no specific environmental precipitant for vision loss in LHON mutation carriers has been clearly identified [**2**]. Nutritional deficiencies (e.g. vitamin B12 deficiency [**3**]) might also play a role in disease expression through an insufficiency of important metabolic cofactors [**2**]. Patients are strongly advised to moderate their alcohol intake and not to smoke to minimize mitochondrial stress [**3**]. Various other systemic illnesses, medications, and toxins have been proposed as triggers for vision loss in the setting of LHON mutations [**2**]. The gender could also result from a combination of subtle anatomic, hormonal, and/ or physiologic variations between males and females [**1**].

## Material and methods

**Case Report**

A 21-year-old Caucasian male was referred to our Department of Ophthalmology for sudden decreased visual acuity, initially in the right eye, followed after two weeks by the left eye. The patient was treated in another medical service with methylprednisolone intravenously followed by oral therapy, but without a favorable clinical response. He was addressed to our clinic, one month after the sudden onset of the visual loss in the right eye. The patient had no medical history of diseases.

On ophthalmologic examination, he had a visual acuity of 20/ 400 in both eyes. The intraocular pressure of the right and left eye was 20 mmHg GAT and 18 mmHg GAT, respectively. The anterior segment of both eyes had a normal appearance on slit-lamp examination and normal direct and consensual pupillary light reflexes and the absence of a relative afferent pupillary defect were also noted. 

**Fig. 1:**
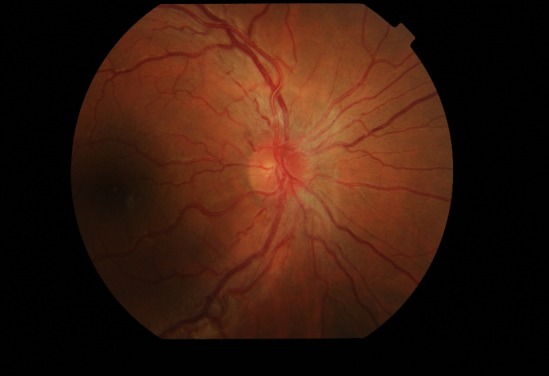
Fundus examination of the right eye at presentation

**Fig. 2:**
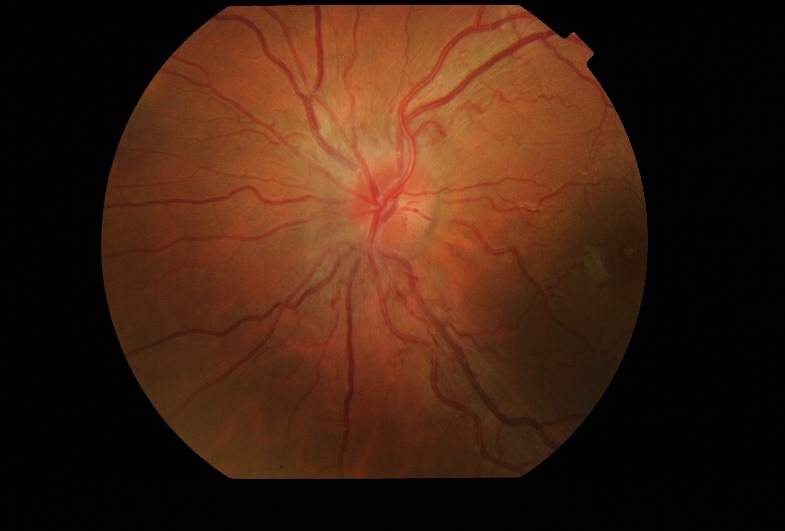
Fundus examination of the left eye at presentation

Fundus examination (**Fig. 1**,**2**) demonstrated bilateral protruding, hyperemic, with blurred margins in the nasal quadrant papilla and reduced excavation, tortuous vessels, peripapillary telangiectasias. 

**Fig. 3:**
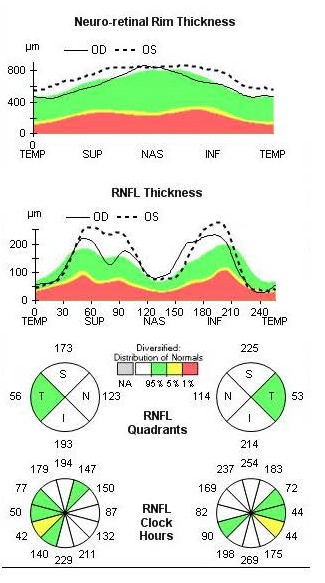
Bilateral increase of the RNFL thickness at presentation

**Fig. 4:**
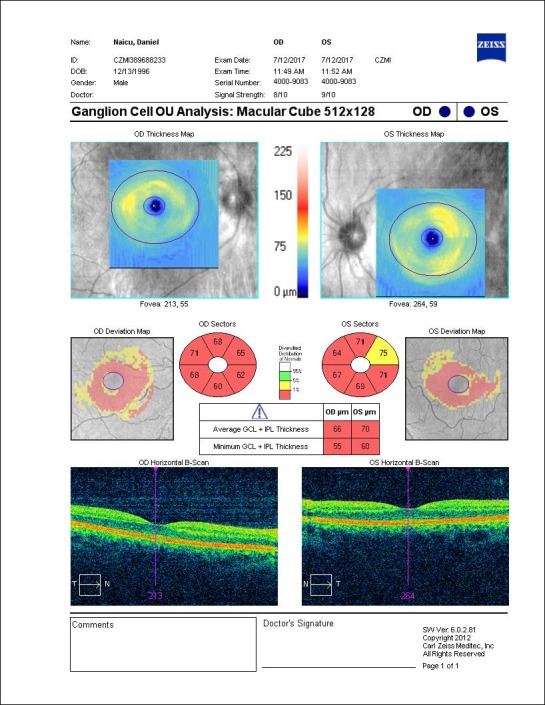
Severely thinned GCL-IPL complex at presentation

The OCT revealed bilateral increase of the RNFL thickness (**Fig. 3**) and GCL-IPL complex severely thinned (**Fig. 4**).

Based on the anamnesis, the ophthalmological examination, and the OCT examination, the first stage diagnosis was optic neuritis in both eyes. In order of importance, different causes of bilateral papillary edema in a young adult [**4**] were taken into consideration. Intracranial expansive processes, as tumors, hemorrhages, hematomas and pseudotumor cerebri, were excluded by magnetic resonance imaging (MRI) scan. Nearby inflammatory processes, such as sinus or dental, were excluded from the anamnesis and MRI scan. Infectious processes, like syphilis, were excluded by paraclinical investigations. We excluded arteritic [**5**] and non-arteritic [**6**] anterior ischemic optic neuropathies due to absent blood dyscrasias in our patient and the age segment of which he is part. We have also excluded multiple sclerosis and optic nerve and orbit tumors that rarely cause bilateral damage. Last, but not least, we considered LHON, which was confirmed by genetic testing three weeks later.

**Fig. 5:**
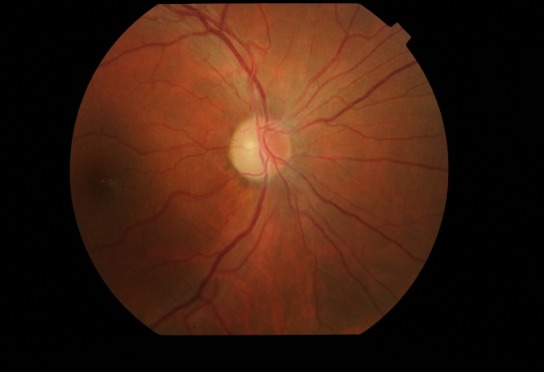
Fundus examination of the right eye at initiation of treatment with idebenone

**Fig. 6:**
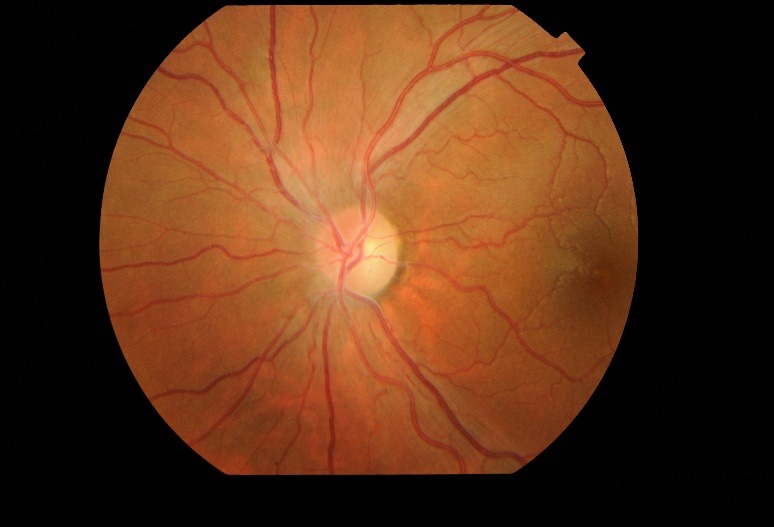
Fundus examination of the left eye at initiation of treatment with idebenone

**Fig. 7:**
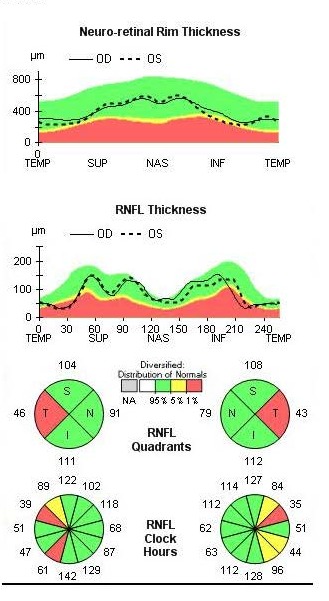
Normal RNFL thickness, except for the temporal quadrants where the thickness is decreased, at initiation of the treatment with idebenone

**Fig. 8:**
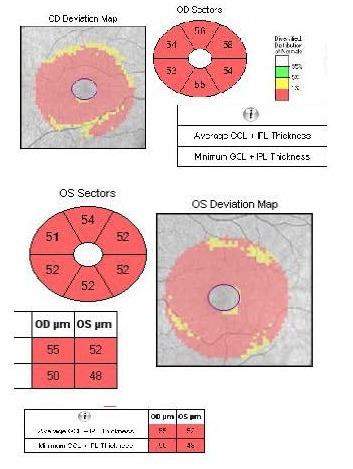
Severely thinned GCL-IPL complex at initiation of treatment with idebenone

**Fig. 9:**
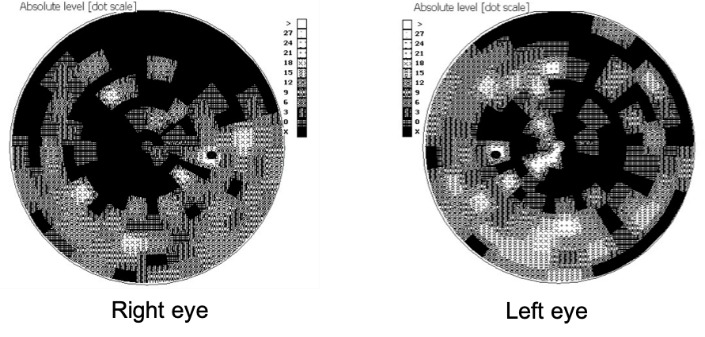
Scotoma in the centrocecal area at initiation of treatment with idebenone

Subsequently, the administrative procedures for obtaining the treatment with idebenone were initiated and treatment was started four months later after the presentation. The patient administrated idebenone as recommended by the producer (300 mg T.I.D.). At the time of initiation of idebenone treatment, the visual acuity decreased to counting fingers (CF) in both eyes, the intraocular pressure of the right and left eye was 19 mmHg GAT, and pupillary reflexes were normal. Fundus examination at initiation of treatment (**Fig. 5**,**6**) revealed plane papilla, with net contour, pale and small excavation at both eyes. The vessels had normal caliber and route. The OCT examination of the optic nerve revealed a normal thickness of RNFL (**Fig. 7**), except for the temporal quadrants, where the thickness was low. Regarding the GCL-IPL complex (**Fig. 8**), an emphasis of the thickness decrease compared to the moment of presentation was noticed. Perimetry at initiation of the idebenone treatment (**Fig. 9**) showed an overall reduction in the sensitivity of the nerve fibers with absolute scotoma in the centrocecal area.

**Fig. 10:**
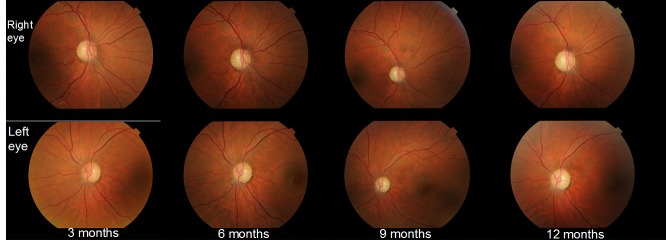
Fundoscopy at three, six, nine, and twelve months from initiation of the treatment with idebenone

**Fig. 11:**
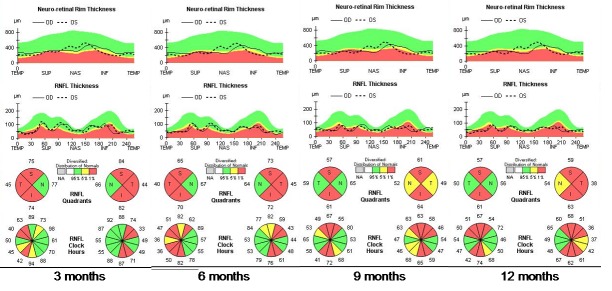
Progression of atrophy of the optic nerve in all quadrants, except for the nasal fibers that have been spared, at three, six, nine and twelve months from initiation of the treatment with idebenone

**Fig. 12:**
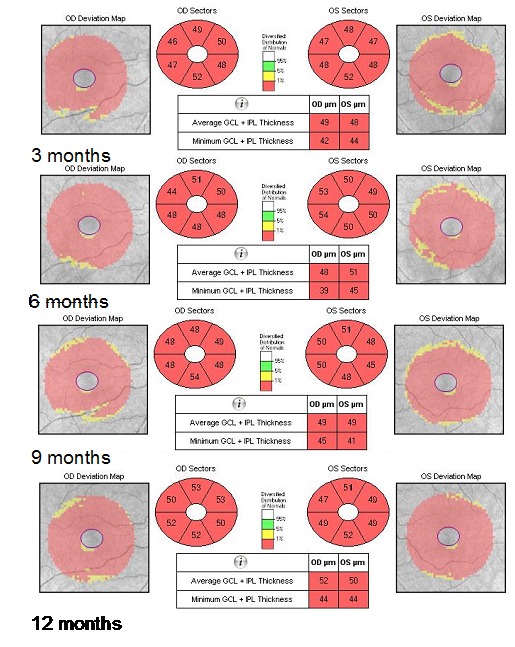
Severely thinned GCL-IPL complex, at three, six, nine, and twelve months from initiation of the treatment with idebenone

**Fig. 13:**
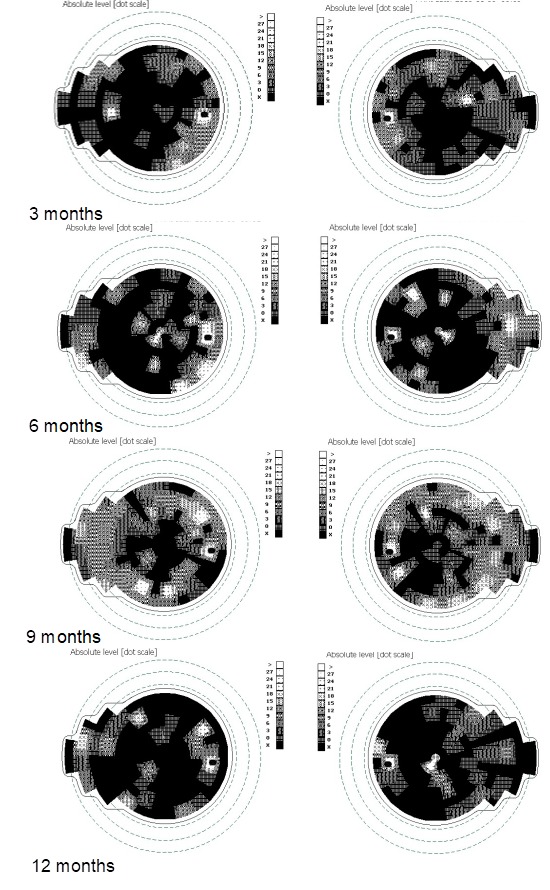
Persistence of the centrocecal scotoma and its reduction in periphery, at three, six, nine, and twelve months from the initiation of the treatment with idebenone

From the initiation of treatment, the patient was reevaluated at every three months (at three, six, nine, and twelve months). None of the reevaluations showed any improvement of the visual acuity. The fundoscopy (**Fig. 10**) aspect remained the same from the initiation of treatment. Three months after treatment initiation, the OCT assessment of the optic nerve revealed progression of atrophy in all quadrants, except for the nasal quadrants, which were spared. At six, nine, and twelve months, we found a stationary look (**Fig. 11**). The OCT assessment of the GCL-IPL complex (**Fig. 12**) revealed a stable appearance. As it was observed, there was an improvement at the perimetric evaluation (**Fig. 13**), the discreet reduction of the scotoma in periphery, but unnoticed by the patient.

## Discussions

We considered we could surprise some particular elements of LHON, such as the transmission by maternal inheritance, the selective vulnerability of the optic nerve and the retinal ganglion cells, the pattern of nerve fiber damage, the absence of relative afferent pupillary defect, gene phenotyping and the decreased response rate and how idebenone works.

**Fig. 14:**
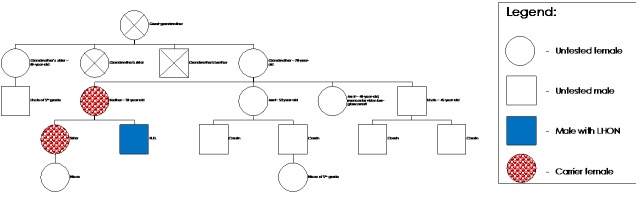
Pedigree

LHON is caused by a variety of maternal transmission mutations with variable penetration [**1**]. Therefore, we considered necessary genetic testing of the mother and sister of the patient. As we could observe, both were healthy carriers of the 3460 mutation and are periodically evaluated to detect early signs of the disease (**Fig. 14**).

The ocular pathology in LHON is limited to the retinal ganglion cell layer with the sparing of the retinal pigment epithelium and photoreceptor layers [**1**]. A number of pathologic factors have been implicated, including a reduction for ATP being produced by the mitochondrial respiratory chain, impaired glutamate transport, and increased levels of reactive oxygen species production, all of which ultimately trigger retinal ganglion cell death via an apoptotic mechanism [**1**]. Neurologic abnormalities such as postural tremor, peripheral neuropathy, nonspecific myopathy, and movement disorders have been reported to be more common in individuals with LHON than in the general population. A multidisciplinary approach for those affected individuals with extraocular neurologic features should be considered [**1**].

The selective vulnerability of the optic nerve and the retinal ganglion cells (RGCs) that comprise it, to mitochondrial dysfunction in LHON, may relate to uneven energy demands along each RGC axon [**2**]. Histochemical studies of RGC axons have shown mitochondrial clustering in areas with a high density of repolarization sodium-potassium membrane pumps, and an abrupt decrease in mitochondrial numbers was seen posterior to the lamina cribrosa where myelination begins and energy-efficient saltatory conduction occurs [**2**]. This uneven distribution of mitochondria suggests an increased energy requirement and special vulnerability of the unmyelinated retinal and prelaminar portions of the RGC axons to bioenergetic failure in LHON [**2**].

**Fig. 15:**
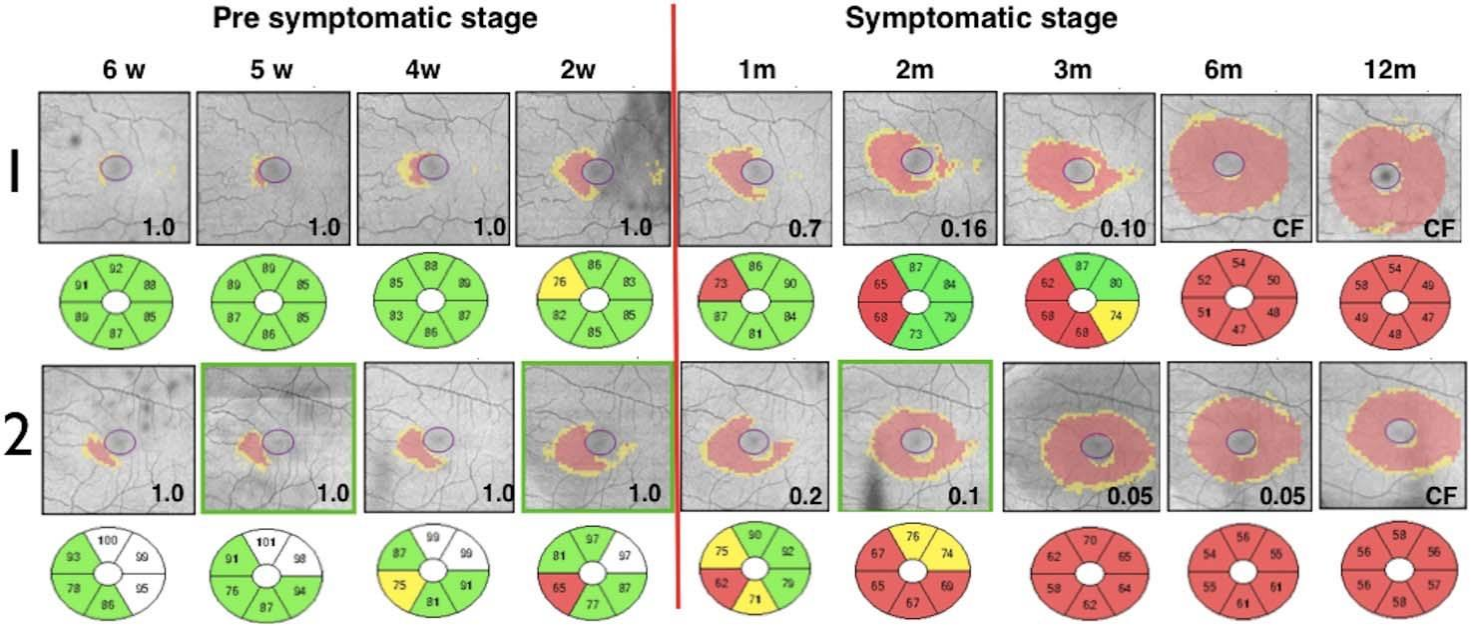
Pattern of CGL-IPL complex damage [**7**]

Major advancements in understanding LHON were possible after the introduction of the OCT [**7**]. Regarding the GCL-IPL complex, macular OCT revealed selective loss of the GCL-IPL complex thickness in parallel with the loss of the temporal peripapillary RNFL thickness [**8**]. The natural history of GCL-IPL thinning follows a specific pattern of reduction, reflecting the anatomical course of papillomacular fibers [**7**]. GCL-IPL thinning was detectable in the deviation map during the presymptomatic stage in the inner ring of the nasal sector and then it progressively extended following a centrifugal and spiral pattern (**Fig. 15**) [**7**].

**Fig. 16:**
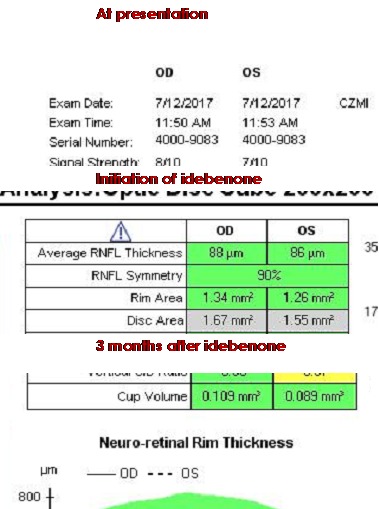
Pattern of RNFL damage

In terms of the RNFL, thickness changes in the early stage of LHON, this showing a specific pattern of early thickening and late thinning (**Fig. 16**) [**7**]. 

The RNFL swelling is likely to depend on the compensatory increase of mitochondrial biogenesis and/ or axonal stasis along the fibers, and this does not allow detection of optic nerve atrophy in the early stages of the disease [**7**]. Thus, in the presymptomatic and early stage of LHON, atrophic evolution is better represented by GC-IPL analysis of the macular sectors [**7**]. Therefore, GC-IPL analysis can detect the optic nerve damage in patients with LHON earlier than RNFL analysis. Instead, during the middle and late stages of the disease (3–12 months), RNFL thickness provides the most reliable information about disease progression. The temporal and inferior quadrants show the earliest involvement of swelling, whereas the superior and nasal quadrants are affected later on [**7**]. The pattern of fiber loss in LHON does not seem to be influenced by the specific mtDNA mutation [**9**].

The absence of relative afferent pupillary defect is another feature of LHON. A relative pupillary defect is caused by an incomplete optic nerve lesion or severe retinal disease. It is highlighted by the swinging flashlight test [**3**]. The presence of melanopsin-containing RGCs was first noted in 1923 when rodless, coneless mice still responded to a light stimulus through pupil constriction, suggesting that rods and cones are not the only light sensitive neurons in the retina [**10**]. They represent a very small subset (~1%) of the retinal ganglion cells [**11**]. Melanopsin retinal ganglion cells resist neurodegeneration due to mitochondrial dysfunction and maintain non-image-forming functions of the eye in these visually impaired patients [**11**]. They contribute to the regulation of pupil size and other behavioral responses to ambient lighting conditions [**10**].

**Fig. 17:**
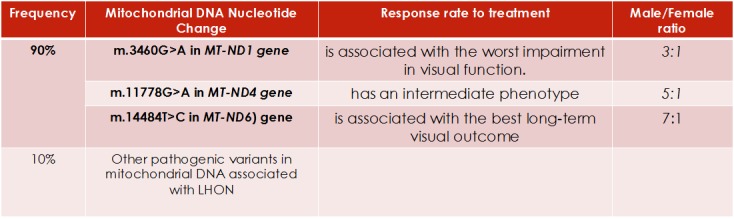
Gene phenotyping and decreased response rate [**1**]

LHON is diagnosed by molecular genetic testing (**Fig. 17**) for one of three common mtDNA pathogenic variants, a multi-gene panel, or complete mtDNA sequencing [**1**]. The table below illustrates the response rate at treatment with idebenone of the three most commented mutations and their rate of occurrence in males and females. Other positive prognostic factors have been identified including an earlier age of onset (< 10 years), a subacute presentation with slow visual deterioration, and a relatively large optic disc [**1**]. Even so, most persons remain severely visually impaired and are within the legal requirements for blind registration [**1**].

**Fig. 18:**
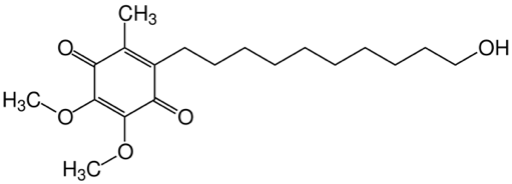
Idebenone

Idebenone (**Fig. 18**), a short-chain benzoquinone, is an anti-oxidant assumed to transfer electrons directly to complex III of the electron transport chain, thereby bypassing complex I, which is affected by all three primary mtDNA mutations causing LHON, and restoring cellular ATP generation [**12**]. According to this biochemical mode of action, idebenone may reactivate viable, but inactive RGCs in LHON patients [**12**]. Clinical safety and efficacy of idebenone in LHON have been assessed in one double blind, randomized, placebo-controlled study (RHODOS) [**12**]. A greater proportion of those in the treated group recovered vision compared with the untreated group, and the most consistent factor associated with visual recovery was an early initiation of treatment during the acute phase of the disease process [**1**].

We noticed case particularities as it follows:

• The rapid evolution of the visual loss, within one month – mean: 6 months [**9**].

• The sudden onset of the visual loss of the second eye, only two weeks apart from the first eye – mean: 6 to 8 weeks [**2**].

• The lowest prevalence of the 3460 mitochondrial DNA mutation.

• It is hard to assess the evolution under idebenone treatment, since the 3460 mutation has a low prevalence, and there is insufficient data about it.

• Since the 3460 mitochondrial DNA mutation, the importance of genetic testing of relatives has the highest penetrability in females.

**Financial Disclosure**

None of the authors has any financial or proprietary interest to disclose.
